# Diagnosis progress of carcinoma of unknown primary

**DOI:** 10.3389/fonc.2024.1510443

**Published:** 2024-11-26

**Authors:** Yun Qiao, Mei Wang, Kaiyuan Hui, Xiaodong Jiang

**Affiliations:** ^1^ Department of Oncology, Lianyungang Clinical College of Nanjing Medical University, Lianyungang, China; ^2^ Department of Oncology, The Affiliated Lianyungang Hospital of Xuzhou Medical University, Lianyungang, China

**Keywords:** carcinoma of unknown primary, gene expression profiling, genetic testing, personalized treatment, traceable diagnosis

## Abstract

Carcinoma of unknown primary (CUP) is a common and complex type of tumor in clinical practice, where the primary site cannot be determined through conventional diagnostic methods, posing significant challenges for clinical diagnosis and treatment. In recent years, advancements in gene expression profiling and genetic testing technologies have provided new perspectives for CUP research, driving progress in this field. By analyzing gene expression profiles, researchers can more effectively identify the tissue origin of tumors, thereby improving diagnostic accuracy. At the same time, the potential application of genetic testing is continuously being explored, offering new possibilities for personalized treatment. This article aims to discuss the latest advancements in the diagnosis of CUP, analyze the importance of gene expression profiling and genetic testing in tumor origin identification and their clinical applications, and summarize current research progress and future research directions, with the goal of providing a theoretical basis for the early diagnosis and treatment of CUP.

## 1 Introduction

Carcinoma of unknown primary (CUP) refers to a clinically diagnosed metastatic tumor, but the type of primary tumor cannot be found through a series of imaging and pathological examinations. The incidence of CUP has been increasing year by year, accounting for approximately 3%-5% of all malignant tumors, making it the eighth most common tumor, with a mortality rate ranking fourth (1). CUP is a group of metastatic malignant tumors with significant heterogeneity and highly malignant phenotypes, with a median overall survival of 2.7-11 months and a one-year survival rate of 15%-20% (2). Although the exact pathogenesis of CUP has not been fully elucidated, it can occur in different age groups and genders, with a higher incidence in male patients, particularly in the middle-aged and elderly population (3). Due to the lack of a clear primary site, doctors often face numerous difficulties in diagnosis. In clinical practice, histopathological examination remains the primary means of diagnosing CUP, but various factors such as insufficient sampling, tumor heterogeneity, differences in tissue antigenicity, and subjective interpretation by observers can lead to biases in pathological diagnosis. Traditional pathological and imaging techniques have a diagnostic rate of only 20%-30% for determining the primary site of CUP (4). In recent years, although the development of fluorodeoxyglucose positron emission tomography/computed tomography (18F-FDG PET/CT) has improved the detection rate of CUP patients, it is still difficult to detect small lesions and those with low activity; while the elevation of various tumor markers in the blood is somewhat related to the type of primary tumor, both specificity and sensitivity are not very high (5). The vast majority of patients also have poor prognoses due to the lack of effective treatment methods resulting from unclear diagnoses.

In recent years, advancements in gene expression profiling and genetic testing technologies have provided new ideas for the diagnosis of CUP. These technologies can help determine the origin of tumors by analyzing the genetic characteristics of tumor cells, thus providing a basis for personalized treatment (6). Therefore, exploring the application of gene expression profiling and genetic testing in the diagnosis of CUP has significant clinical implications.

## 2 Pathological features of CUP

### 2.1 Pathological classification and histological characteristics

CUP is a common type of malignant tumor in clinical practice, with complex and diverse pathological features. Based on histological characteristics, CUP can be classified into various types, including adenocarcinoma, squamous cell carcinoma, and undifferentiated carcinoma. Studies have shown that adenocarcinoma is the most common type in CUP, followed by squamous cell carcinoma and neuroendocrine tumors (7). Histologically, CUP exhibits diverse cell morphology, often characterized by significant cellular atypia, increased nuclear-cytoplasmic ratio, and increased mitotic figures. Additionally, through immunohistochemical staining, the pathological classification of CUP can be further refined, with commonly used markers including CK7, CK20, and TTF-1, which help determine the possible origin and type of the tumor (8). Although the exact origin of CUP remains unclear, recent studies have indicated that the molecular characteristics and genomic changes of tumors play an important role in their pathological classification, providing new diagnostic ideas and therapeutic targets for clinical practice (9).

### 2.2 Common clinical manifestations and imaging features

The clinical manifestations of CUP patients are often subtle, typically lacking specific symptoms in the early stages. As the disease progresses, systemic symptoms such as weight loss, fatigue, decreased appetite, and pain may appear (10). Imaging examinations play an important role in the diagnosis of CUP, with CT, MRI, and PET-CT helping doctors identify the metastatic sites and characteristics of the lesions. CT scans often show multiple lymphadenopathy and metastatic lesions in the liver or lungs, while MRI provides clearer images of soft tissue masses (11). Additionally, PET-CT has high sensitivity in distinguishing between benign and malignant lesions, helping to identify potential primary sites (12). Although imaging examinations are significant for the diagnosis of CUP, in some cases, pathological biopsy is still needed to confirm the diagnosis and formulate appropriate treatment plans (13).

## 3 Research progress on tumor tissue origin

### 3.1 Traditional theories of tumor tissue origin

The origin of tumor tissue has always been an important topic in cancer research. Traditional theories of tumor origin mainly focus on genetic mutations in cells and changes in the tumor microenvironment. These theories suggest that tumor development is usually due to cumulative mutations in the genome of cells, leading to a loss of normal growth regulatory mechanisms. This theory emphasizes the clonal selection and evolutionary process of tumor cells, positing that tumor cells evolve from normal cells through multiple stages of mutation and selection, ultimately forming a population of tumor cells with invasive and metastatic capabilities. Furthermore, the extracellular matrix, immune cells, and other supportive cells in the tumor microenvironment are also believed to play important roles in tumor occurrence and development (14, 15). Although traditional theories have achieved some success in explaining the mechanisms of many tumors, there remain numerous unresolved mysteries, such as the heterogeneity of different tumor types and the complexity of the early stages of tumor development (16, 17).

### 3.2 Emerging molecular biological perspectives

In recent years, with the rapid development of molecular biology and genomics technologies, research on tumor tissue origin has begun to explore deeper molecular mechanisms. This emerging perspective not only focuses on genetic mutations in tumor cells but also emphasizes the interactions between tumor cells and their microenvironment. For example, studies have shown that the DNA methylation patterns of tumor cells can serve as biological markers for their tissue origin and type (14). Through high-throughput sequencing technology, researchers can identify specific methylation characteristics of different tumor types. Additionally, the molecular classification of tumor tissues is continuously evolving, with research finding that the occurrence of certain tumors is related to specific cell types or tissue sources, suggesting that treatment strategies may need to be personalized based on different molecular characteristics (18). By integrating multi-omics data, researchers are gradually revealing the complex networks of tumor occurrence, striving to find new therapeutic targets and early diagnostic methods at the molecular level (19). This multidimensional research strategy provides new insights into understanding the origin and development of tumors.

## 4 Application of gene expression profiling in CUP

### 4.1 Basic concept of gene expression profiling

Gene expression profiling refers to the overall expression levels of all genes in a cell or tissue under specific conditions. Through high-throughput technologies such as microarray chips and RNA sequencing, researchers can obtain a large amount of gene expression data. These data not only reveal the activity status of genes but also provide important information about cellular functions, developmental processes, and pathological states. In cancer research, the application of gene expression profiling is particularly widespread, especially in the diagnosis, prognosis, and treatment selection of cancer. For CUP patients, the analysis of gene expression profiling can help identify the origin of tumors, thereby guiding clinical decision-making. Studies have shown that gene expression profiling can infer the tissue origin of tumors by comparing the expression characteristics of patient tumor samples with those of known primary tumors, thus providing a basis for personalized treatment (20, 21).

### 4.2 Case analysis of tumor origin identification through gene expression profiling

Multiple research cases have shown that gene expression profiling has significant application value in identifying the tumor origin of CUP (Cancer of Unknown Primary). For example, one study successfully identified the tumor origins of several CUP patients using a 90-gene expression profiling method, revealing that the tumors of these patients shared similar gene expression characteristics with specific primary tumors, thus providing potential therapeutic targets and prognostic information for clinical practice (22). Another study integrated gene expression data with clinical information, discovering that specific gene expression patterns were associated with patient survival, further emphasizing the importance of gene expression profiling in the management of CUP patients (23). Additionally, gene expression profiling can be combined with other molecular features such as DNA methylation and mutation information to provide a more comprehensive analysis of tumor characteristics, thereby improving the accuracy of tumor origin identification (24, 25). These findings indicate that gene expression profiling not only offers new perspectives for the biological research of CUP but also provides important guidance for clinical practice.

## 5 Current status and development of genetic testing technologies

### 5.1 Commonly used genetic testing methods (e.g., NGS, PCR)

Genetic testing technologies have made significant progress in recent years, particularly with the application of high-throughput sequencing technology (Next-Generation Sequencing, NGS) and Polymerase Chain Reaction (PCR). NGS technology, known for its speed and accuracy, is widely used for comprehensive genomic analysis, enabling the sequencing of a large number of genes in a short time, greatly advancing the development of personalized medicine. On the other hand, PCR, as a classic genetic testing method, continues to play an important role in many clinical applications, especially in detecting specific gene mutations and assessing microsatellite instability (MSI) status. The combined use of PCR and NGS enhances the sensitivity and specificity of testing (26). Furthermore, with the development of CRISPR technology, the ability to edit and regulate genes has also provided new ideas for genetic testing, further promoting the advancement of precision medicine.

### 5.2 Specific applications of genetic testing in CUP diagnosis

In the early diagnosis of cancer, CUP cases pose significant challenges to clinicians. Genetic testing technologies, especially NGS, have shown great potential in the diagnosis of CUP. By conducting genomic analysis on tumor samples, researchers can identify specific gene mutations and expression patterns, thereby inferring the possible origins of the tumors. For instance, one study successfully provided tissue origin diagnostic information for CUP patients using a gene expression ranking algorithm (TOD-CUP), significantly improving diagnostic accuracy (27). Additionally, a computational framework based on RNA sequencing has been proposed to trace the tissue origin of tumors, providing new ideas and methods for the molecular management of CUP (28). These cases demonstrate that the application of genetic testing technologies in CUP diagnosis not only improves diagnostic accuracy but also provides important evidence for subsequent treatment decisions, facilitating the implementation of personalized treatment. With continuous technological advancements, genetic testing will play an increasingly important role in the diagnosis of CUP and other complex diseases.

### 5.3 Prognostic assessment and monitoring

Genetic testing also plays a crucial role in the prognostic assessment and monitoring of diseases. By analyzing gene variations associated with disease progression, doctors can more accurately predict patient prognosis. Genetic testing results for certain cancer patients can be used to assess tumor aggressiveness and response to treatment, helping doctors develop more effective follow-up plans (29, 30). In summary, genetic testing not only provides important prognostic assessment tools for clinicians but also lays the foundation for long-term health monitoring of patients, promoting the development of precision medicine.

## 6 Future research directions and challenges

### 6.1 Multi-omics research combining genomics and clinical data

Future research directions should focus on integrating genomics with clinical data to conduct multi-omics studies. This integration can provide more comprehensive information for early cancer diagnosis, prognostic assessment, and treatment planning (31). In recent years, with the rapid development of genomic technologies, particularly in cancer genetic testing and biomarker discovery, the integration of clinical data has become particularly important. For example, studies have shown that integrating genomic data with clinical information can significantly improve cancer identification rates and treatment precision. In the study of CUP, the application of genomics can not only help identify tumor origins but also reveal molecular characteristics of tumors, providing a basis for personalized treatment (32). Additionally, multi-omics research can explore the interactions between different omics data through network biology methods, thereby revealing the complex mechanisms of tumor development (33). Therefore, future research should emphasize the integration of multi-omics data to advance cancer research to a deeper level.

### 6.2 Prospects of personalized treatment in CUP diagnosis and treatment

Personalized treatment shows great promise in the diagnosis and treatment of CUP. As a special type of cancer, CUP has diverse clinical manifestations and complex etiologies, making traditional treatment methods often ineffective. In recent years, with the rise of personalized medicine concepts, treatment strategies for CUP patients have gradually shifted towards personalization. For example, genomic analysis can identify specific gene mutations associated with tumors, allowing for the development of more targeted treatment plans for patients (34). Furthermore, the application of emerging technologies such as machine learning has made it possible to conduct in-depth analyses of patients’ genomic data, thereby improving the predictive ability for treatment responses (35). At the same time, personalized medicine emphasizes the overall health status and lifestyle of patients, and treatment plans developed after considering these factors can better meet patients’ needs and improve their quality of life. Therefore, future research should further explore the application of personalized medicine in CUP, promoting its implementation and development in clinical practice.

## 7 Conclusion

In this review, we have explored the diagnostic progress of CUP, particularly the key roles of gene expression profiling and genetic testing in CUP. With advancements in molecular biology technologies, genetic testing provides us with unprecedented tools to identify and understand the biological characteristics of these complex tumors. Research indicates that gene expression profiling can not only help determine tumor origins but may also reveal potential targets for personalized treatment. This shift not only advances research in oncology but also offers new hope for clinical practice.

Looking ahead, we need to integrate various research findings in the management of CUP to form a more comprehensive theoretical framework and explore new clinical application strategies. This includes optimizing existing testing technologies and focusing on the introduction of emerging technologies, such as liquid biopsies, which may further enhance our understanding and management capabilities regarding CUP. Additionally, the conduct of clinical trials will be a key step in validating the effectiveness of these new ideas and technologies.

In conclusion, gene expression profiling and genetic testing show great potential in the diagnosis of CUP, but to maximize their clinical application, we still need to overcome numerous challenges. Through interdisciplinary collaboration and ongoing research efforts, we hope to provide more effective diagnostic and therapeutic solutions for CUP patients, improving their prognosis.
